# Closing the health equity gap: evidence-based strategies for primary health care organizations

**DOI:** 10.1186/1475-9276-11-59

**Published:** 2012-10-13

**Authors:** Annette J Browne, Colleen M Varcoe, Sabrina T Wong, Victoria L Smye, Josée Lavoie, Doreen Littlejohn, David Tu, Olive Godwin, Murry Krause, Koushambhi B Khan, Alycia Fridkin, Patricia Rodney, John O’Neil, Scott Lennox

**Affiliations:** 1 University of British Columbia, School of Nursing, T201 – 2211 Wesbrook Mall, Vancouver, British Columbia, V6T 2B5, Canada; 2 University of Northern British Columbia, School of Health Sciences, 3333 University Way, Prince George, British Columbia, Canada; 3 Vancouver Native Health Society, 440 East Hastings Street, Vancouver, British Columbia, Canada; 4 Prince George Division of Family Practice, Prince George, British Columbia, Canada; 5 Central Interior Native Health Society, 1140 4th Avenue, Prince George, British Columbia, Canada; 6 Simon Fraser University, Faculty of Health Sciences, 8888 University Drive, Burnaby, British Columbia, Canada; 7 University of British Columbia Centre for Health Services and Policy Research, 201-2206 East Mal, Vancouver, British Columbia, Canada

**Keywords:** Primary health care, Health equity, Health inequity, Marginalized populations, Vulnerable populations, Aboriginal people, Structural violence, Trauma-informed care, Qualitative research, Ethnographic methods

## Abstract

**Introduction:**

International evidence shows that enhancement of primary health care (PHC) services for disadvantaged populations is essential to reducing health and health care inequities. However, little is known about how to enhance equity at the organizational level within the PHC sector. Drawing on research conducted at two PHC Centres in Canada whose explicit mandates are to provide services to marginalized populations, the purpose of this paper is to discuss (a) the key dimensions of equity-oriented services to guide PHC organizations, and (b) strategies for operationalizing equity-oriented PHC services, particularly for marginalized populations.

**Methods:**

The PHC Centres are located in two cities within urban neighborhoods recognized as among the poorest in Canada. Using a mixed methods ethnographic design, data were collected through intensive immersion in the Centres, and included: (a) in-depth interviews with a total of 114 participants (73 patients; 41 staff), (b) over 900 hours of participant observation, and (c) an analysis of key organizational documents, which shed light on the policy and funding environments.

**Results:**

Through our analysis, we identified four key dimensions of equity-oriented PHC services: inequity-responsive care; trauma- and violence-informed care; contextually-tailored care; and culturally-competent care. The operationalization of these key dimensions are identified as 10 strategies that intersect to optimize the effectiveness of PHC services, particularly through improvements in the quality of care, an improved 'fit' between people's needs and services, enhanced trust and engagement by patients, and a shift from crisis-oriented care to continuity of care. Using illustrative examples from the data, these strategies are discussed to illuminate their relevance at three inter-related levels: organizational, clinical programming, and patient-provider interactions.

**Conclusions:**

These evidence- and theoretically-informed key dimensions and strategies provide direction for PHC organizations aiming to redress the increasing levels of health and health care inequities across population groups. The findings provide a framework for conceptualizing and operationalizing the essential elements of equity-oriented PHC services when working with marginalized populations, and will have broad application to a wide range of settings, contexts and jurisdictions. Future research is needed to link these strategies to quantifiable process and outcome measures, and to test their impact in diverse PHC settings.

## Introduction

Despite Canada’s commitment to provide high quality health care, health inequities remain a pressing concern. Of particular concern are the persistent and growing health and health care inequities affecting marginalized populations [1-5]. In Canada, increasing homelessness; social exclusion experienced by those living with chronic mental illness or substance use; violence against women; and systemic discrimination toward Aboriginal people^a^ and new immigrants are instances of systemic health and social inequities that can be addressed through equity-oriented primary health care (PHC)^b^ interventions, particularly when linked to structural changes and policy shifts.

As reiterated by the World Health Organization (WHO), one of the most efficient ways of ‘closing the equity^c^ gap’ within a population is to address the health and health care needs of those most disadvantaged [6]. International evidence continues to show that enhancement of PHC services for disadvantaged populations is one of the most important means of reducing inequities [7-9]. Repeated calls for PHC renewal are based on solid evidence that a strong PHC foundation leads to improved population health outcomes, including reduced risk and effects of acute and chronic conditions; reduced use of emergency services; lower rates of preventable hospital admissions; and lower overall health care utilization [6,7,10]. Conversely, when PHC is not accessible or effective, people delay seeking help, rely on emergency care, and lose the benefits of continuity of care [3,11,12]. These issues contribute to the increasing importance of the PHC sector in enhancing efforts to orient services toward marginalized populations where the greatest gains in redressing inequities can be achieved [7,9,13,14]. Broad-based PHC approaches and interventions – that integrate accessible, high quality, responsive services *with* structural and policy changes to improve people’s access to the social determinants of health – may therefore be one of the most effective means of working towards greater equity.

In this article, the terms ‘marginalized,’ ‘vulnerable’ or ‘disadvantaged’ refer to the conditions and processes by which particular populations are affected by structural inequities and structural violence in ways that result in a disproportionate burden of ill health and social suffering. We place an emphasis on marginalizing conditions to suggest that particular populations are not inherently marginalized, rather, it is the marginalizing conditions that create and sustain inequities. Structural inequities refer to how policies and practices embedded in systems such as social welfare, economic, justice and health care operate to produce inequitable distribution of the determinants of health [15]. Structural violence is increasingly seen in public and population health as a major determinant of the distribution and outcomes of health inequities and is defined as “a host of offensives against human dignity: extreme and relative poverty, social inequalities ranging from racism to gender inequality, and the more spectacular forms of violence” [15]^(p.8)^. Inequities are structural because they are embedded in the political and economic organizations of our social world, and they are violent because they cause injury to people [15]. For example, discrimination, individual and institutionalized racism, poverty, and social exclusion are consequences of structural inequities and structural violence, and have tangible effects on health status and access to health care [3,16-20]. The health of Aboriginal people in Canada serves as a case in point. Despite improvements in recent years, inequities persist on virtually every measure of health and social status, for example, lower life expectancy, decreased access to health services, and disproportionately high rates of preventable, chronic and acute health conditions [1,4,21,22].

Focusing on the health effects of inequities necessitates attention to the concept of trauma. Trauma is increasingly used to frame the health, social, and psychological effects of structural inequities and structural violence [21,23-35]. Research shows that trauma histories are highly prevalent among marginalized populations [24]. Traumatizing experiences include, for example, discrimination and social exclusion, poverty, emotional abuse, physical violence, sexual assault, torture and war. Traumatizing experiences can be historical and intergenerational such as the experiences of Aboriginal people in Canada. Despite mounting evidence linking trauma with negative health effects (e.g., chronic pain, problematic substance use, mental health issues) [25,32,36,37], little is known about how to address trauma in the context of PHC service delivery to marginalized populations.

Research on PHC delivery highlights several persistent problems: (i) inverse care (i.e., those who are most marginalized and have the greatest health problems have the least access to care); (ii) fragmentation and under-resourcing of care for marginalized populations; (iii) significant gaps in knowledge concerning how to make services responsive to marginalized populations; and (iv) policy and funding environments inadequate to redress these problems [7]. Thus, despite a proliferation of calls for strategies to enhance the ability of the PHC sector to develop ‘equity-competence’, little is known about (a) key dimensions of equity-oriented services that ought to guide PHC organizations; or (b) the strategies, processes and policies for enhancing the capacity to deliver equity-oriented PHC services, particularly for marginalized populations [7,38-40]. The purpose of this article is to describe the key dimensions of PHC services, and the strategies for operationalizing those dimensions when equity is an explicit goal, and when working with marginalized populations. Drawing on research at two PHC Centres in Canada with explicit mandates to provide services to marginalized populations, we discuss the relevance of these strategies for PHC agencies and organizations that aim to address the intersecting health and social needs of people experiencing systemic inequities. We begin with an overview of the study. We then outline key dimensions of equity-oriented PHC. Next, we identify strategies for operationalizing these dimensions at the patient-provider, organizational, and system levels.

## Methods

### Overview of study

The findings discussed in this article are part of a larger four-year study aimed at (a) extending our understanding of *how* PHC services are provided to meet the needs of people who have been marginalized by systemic inequities, (b) identifying key dimensions of PHC services for marginalized populations, and (c) developing PHC indicators that can account for the quality, process, and outcomes of care when marginalized populations are explicitly targeted. In this paper, we focus on the first two objectives; an analysis of the work leading to the development of PHC indicators has been reported elsewhere [41]. Methodologically, this research is informed by critical perspectives of social justice and equity [42-46]. The critical theories upon which we draw focus attention on the development of practical knowledge that has the potential to disrupt and transform inequitable social relations. A central methodological concern is that individual experiences, including those in health care, need to be interpreted and understood within the context of broad social, political and historical relations. Such interpretations are needed to better address systemic inequities in health service delivery. These theoretical perspectives provided a framework that guided our methodological approaches and analytical perspectives.

Approval to conduct this study was provided by two university research ethics boards and by the PHC agencies. Using a mixed methods ethnographic design, the research was conducted at two urban PHC Centres in western Canada whose explicit mandates are to provide PHC services to Aboriginal and non-Aboriginal populations experiencing major social and economic inequities. Both have been in operation since the early 1990s, and are located in the inner city areas of two cities recognized as among the lowest socio-economic neighbourhoods in Canada.

The Centres’ combined patient population is 5,500; a high proportion identify as Aboriginal people, and the majority live in poverty and experience social exclusion, racialization and discrimination on a daily basis. Many suffer the consequences of colonization, including the historical trauma of displacement, forced attendance at residential schools, limited educational and employment opportunities on reserves, the disruption of family and communities, the eradication of language and culture, and the ongoing trauma of race-based violence, and discrimination (e.g., unemployment). Many of the patients reside in single room occupancy hotels, have significant mental health and substance use issues, and other stigmatizing health conditions, including HIV/AIDS. Almost all of the patients have experienced inter-related traumas stemming from violence, childhood neglect or abuse, or sexual exploitation. Many, however, reported a strong sense of community connection and belongingness as people residing in both inner city areas.

To respond to people’s intersecting health and social needs, the services at each Centre are organized around a PHC clinic staffed by physicians, nurses, nurse practitioners, social workers, substance use counsellors, and outreach workers, and include on-site and outreach programs. To different degrees, Indigenous approaches to health and healing are integrated, such as the employment of Aboriginal Elders who provide counselling and support to both Aboriginal and non-Aboriginal patients.

### Data collection

The ethnographic approaches we used are typically under-utilized in medical and health services research and, therefore, represent innovative qualitative approaches applied to the study of health equity. Observational and interview data were collected at the Centres primarily by the principal investigators, who are experienced ethnographic researchers. In-depth interviews were conducted with a total of 114 patients and staff, including: (a) individual interviews with 62 patients, and three focus groups with 11 patients (n = 73 patients), and (b) individual interviews with 33 staff, and an additional eight staff who participated in focus groups (n = 41 staff). An analysis of key organizational documents was also completed to shed light on policy and funding environments. In addition, in-depth interviews were conducted with two decision-makers employed by Health Authorities within which the Centres are located and funded. Written and verbal consent was obtained prior to conducting interviews or observations, and the voluntary nature of participation was reiterated frequently.

The patient interviews were critical to conceptualizing the approaches to care that were most important in the context of people’s lives. Patient interviews focused on their reasons for coming to the Centres, their likes and dislikes about the services provided, their working relationships with staff, comparisons with health care experiences elsewhere, and how services could be improved to better meet their needs. Staff interviews focused on their experiences working at the Centres, those aspects of service delivery that were essential to address inequities, how they supported and facilitated access to health and social services in ways that influenced patients’ overall quality of life, and how continuity of care was established with patients who might otherwise ‘fall through the cracks.’

Participant observation involved over 900 hours of intensive immersion, which was essential to developing knowledge about equity-oriented PHC services grounded in the everyday complexities of clinical practice. Participant observation was conducted as unobtrusively as possible in the waiting rooms, at the reception desks, in team meetings, and during clinical interactions between patients and providers. The focus of observations was on: (i) the general organizational milieu at the Centres (e.g., the use of spaces to create a welcoming environment, staff approaches with patients in the waiting room, how patients were greeted at the reception desk, how conflict was dealt with, and the strategies used to prioritize patients); and (ii) the staff members’ patterns of interacting, relating, and working with patients and with one another, including *how* they attended to patients, *what* they paid attention to, and how the socio-economic or cultural contexts of patients’ lives were taken into consideration.

Using purposeful sampling, patients were recruited to reflect a diversity of ages, genders and positive and negative experiences with the Centres. Recruitment to participate in interviews also involved obtaining consent to participate in observations. Several approaches were used in recruitment: the waiting rooms contained posters and flyers about the study, and interested patients were invited to identify themselves to the reception staff, who in turn notified the researchers; during the process of participant observation, researchers informed patients about the study and invited their subsequent involvement; lastly, to invite the participation of diverse patients, staff and patients identified individuals who were known to have varied experiences with the Centres. Observational data provided a context for discussing, clarifying and exploring issues raised in interviews, and vice versa. Among the patients who participated (n = 73), 52% were women, 45% were men, and 3% identified as transgender. Seventy-seven percent self-identified as Aboriginal, 22% as Euro-Canadian, and 1% as members of a visible minority^d^[47]. Ages ranged from 20 to 72 (mean = 45 years old). Of the Centres’ staff who participated (n = 41), 24% were nurses or nurse practitioners, 22% were physicians, 22% were medical office assistants (MOAs) and office managers, 10% were in administrative leadership positions, 7% were social workers, 5% were substance use counsellors, and 10% were other staff including an Elder, an outreach worker, a support worker and a pharmacist.

### Data analysis

An interpretive thematic analysis was conducted using procedures for qualitatively derived data [48-50]. Interview transcripts and observational notes were repeatedly read by the members of the investigative team to identify recurring and contradictory patterns in the data, and possible linkages to theoretical perspectives. A qualitative computer software package (NVivo, QSR International, 2010) was used to organize and code the narrative data. The researchers met regularly during the coding process to assess inter-rater reliability, resolve discrepancies, and ensure consistency. For example, as the code-book was being developed, each interview was coded by at least two experienced researchers. Discrepancies were discussed and addressed as the concepts and themes reflected in the codes were further refined.

As data were collected and analyzed, coding categories were refined. In the final stages, the analysis shifted to a more abstract and conceptual representation of themes and the key dimensions of PHC. Importantly, observational data provided a means of triangulating interview data, adding to the methodological rigour.

The credibility of our analysis, as a criterion for rigour in qualitative research, was continually evaluated by members of our research team, who included experts in ethnographic research, PHC services and health equity, and a community advisory committee comprised of patients, and health care providers external to the Centres. Throughout, the research team held regular meetings with groups of patients and staff to discuss the analytical insights and themes. We used diverse strategies including: co-hosting lunches with peer-support workers, during which we invited patients to provide feedback on the findings; engaging in outreach activities such as community tours with patients and staff to gain further insights; consulting with Aboriginal Elders to ensure their perspectives were taken into account; and consulting frequently with the community advisory committee to seek their input. These stakeholders confirmed that the identified themes reflected in the data resonated with their experiences and interpretations, and that the framework we proposed captured the essential features of equity-oriented PHC. For example, at a lunch hosted for ~ 25 patients, the patients completely took over the discussion of the proposed strategies, emphasizing their importance. One man commented, “I thought I was just coming for lunch, but this was the best meeting ever.” Throughout, an audit trail of analytical insights and decisions was maintained.

## Results and discussion

**Figure 1:**
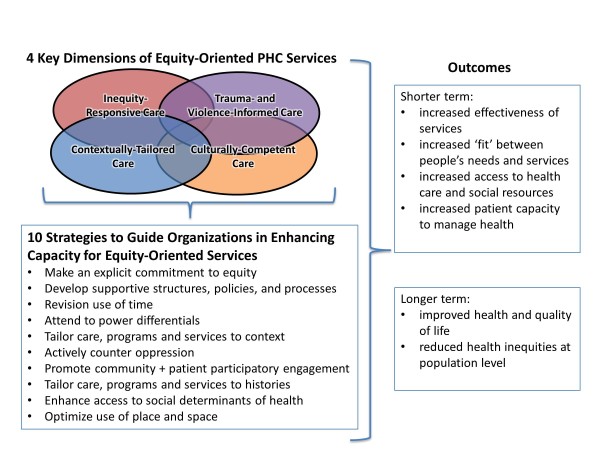
Enhancing equity-oriented PHC delivery.

Through the analysis we identified (a) four key dimensions of equity-oriented PHC services, which are particularly relevant when working with marginalized populations, (b) 10 strategies to guide organizations to enhance their capacity for equity-oriented services, and (c) outcomes related to these dimensions and strategies (see Figure 1).

Conceptually, the four key dimensions provide a framework for understanding the essential elements of equity-oriented PHC services when working with marginalized populations. The dimensions are interrelated and overlapping, and include:

● *Inequity-Responsive Care*: explicitly addressing the social determinants of health as legitimate and routine aspects of health care, often as the main priority.

● *Trauma- and Violence-Informed Care*: recognizing that most people affected by systemic inequities and structural violence have experienced, and often continue to experience, varying forms of violence with traumatic impact. Such care consists of respectful, empowerment practices informed by understanding the pervasiveness and effects of trauma and violence, rather than ‘trauma treatment’ such as psychotherapy.

● *Contextually-Tailored Care*: expanding the concept of patient-centred care to include services that are explicitly tailored to the populations served and local contexts. This may include organizational tailoring to address the local population demographics and social trends (e.g., programs or services addressing HIV, seniors, women’s or men’s issues, support for new immigrants, etc.).

● *Culturally-Competent Care*: taking into account not only the cultural meaning of health and illness, but equally importantly, people’s experiences of racism, discrimination and marginalization and the ways those experiences shape health, life opportunities, access to health care, and quality of life.

The operationalization of these key dimensions is identified in this paper as strategies that intersect to optimize the effectiveness of services, particularly through improvements in the quality of care, an improved ‘fit’ between people’s needs and services, enhanced trust and engagement by patients, and a shift from crisis-oriented care to continuity of care [37,41]. As shown in Figure 1, both patients and providers directly linked these specific strategies to short-term outcomes such as an increase in patients’ capacities to manage their own health, and increased access to resources essential to support health. Both also identified longer-term improvements in health and quality of life, with the potential for reducing health inequities at the population level. These 10 overlapping strategies are discussed below to illustrate their relevance at three inter-related levels: organizational, clinical programming, and patient-provider interactions. These are proposed as principles of equity-oriented services, recognizing that PHC organizations, agencies and practices will have to tailor the extent to which they can implement each strategy in their local context.

### Ten strategies to guide organizations in enhancing capacity for equity-oriented services

1. Explicitly articulate the commitment to equity in the mission, vision and other organizational policy statements

When PHC organizations acknowledge the impact of health and social inequities on health, illness and access to health care, the possibility of organizing and delivering services that foster equity for vulnerable patients and populations is promoted. Key to positioning equity as an explicit mandate is the articulation of related commitments in governance, mission statements, and organizational-level policy statements. This requires strong leadership within the organization, and the continual reflection of mission statements in job postings, staff orientations, staff performance reviews, and related documents and activities. The activities of the organizational leaders are important in reinforcing the organization’s commitment to equity, including for example, the kinds of advocacy and social justice or policy work that leaders engage in within the wider community. One of the Centres’ administrative leaders commented:

"Trying to articulate what our mission and vision and values are….I mean it’s a big piece of work. So you don’t just ignore it once you’ve produced the document. We agonized over those. So we darn well better make sure it’s entrenched in what we do."

At the organizational level, hiring practices need to ensure a match between staff and the organization values, philosophy, and the approaches to care that represent the operationalization of the mission statement. Declaring an organizational commitment to fostering equity can contribute to solidifying an organization’s identity and can serve to attract employees who share similar values regarding PHC and social justice, as noted by this physician: “it’s not a hidden agenda – it’s right out there…it’s part of what you’re getting into.” Working within a PHC organization distinguished by an explicit, organizational commitment to equity can represent a defining feature of practice for direct service providers, as this staff member discussed:

"I think to have it [a commitment to equity] as a shared value is very central to what we do…That’s what’s different, if you’ve ever worked anywhere where people don’t really talk about these things, never mind work on them and try to make it a central core."

At the level of direct clinical care, mission and vision statements also provide a sense of perspective and purpose when progress toward achieving improved health for particular patient-groups is slow or ostensibly backsliding, as a provider explained:

"It is easy to talk about the rhetoric [related to equity], but when you’re here and the patient is, you know, it’s the ninth time you’ve seen them in a month and you’re not getting anywhere. She or he [the organizational leader] is a person who can take you back to the vision and can take you back to, you know, how to see the bigger picture."

Commitments to equity, however, cannot be enacted and will not be sustainable without supportive funding and policy environments.

2. Develop and advocate for structures, policies, and processes to support the enactment of equity

Funding and policy environments that are supportive of equity-oriented aims are needed. This means that leaders must develop appropriate structures, policies and processes within their organizations and advocate within larger contexts for the conditions necessary to achieve these aims. Within the Centres, leaders created an organizational culture that supported interdisciplinary team meetings and the active participation of all team members in planning care, programs, and case management. These activities need to be supported by stable funding arrangements to enhance the ability of the organization to enact a philosophical commitment to equity, and by social policy that enhances access to social determinants of health. Thus, Centre leaders were active at local community, city, provincial and federal levels advocating for adequate social housing, better access to income supports, and allocation of health care funding within their own and other health care organizations (to support, for example, methadone programs and other harm reduction activities).

Efforts to create conditions to support the enactment of equity were also influenced by factors beyond the Centres. For example, leaders negotiated for physicians to be paid by salary rather than fee-for-service to enable their participation beyond direct patient care in interdisciplinary meetings and case conferences; however, such meetings were constantly under threat due to fiscal pressures and questions raised by funders about how clinical staff were using time – pressures that varied with the degree of independence each Centre had in relation to their funding bodies. The Centres varied in the extent to which their contractual funding arrangements permitted them to enact the strategies of equity-oriented care. For example, block-funding arrangements with longer terms permitted greater flexibility at one Centre, whereas at the other, year-to-year and fragmented funding and chronic underfunding limited the range of services in place to address the complexities associated with violence, poverty, homelessness, or substance use. Funding is also needed to provide counselling or debriefing for staff who are dealing with the vicarious effects of structural violence and trauma, as discussed further in strategy eight. A manager explained:

"[Our funders were]…micro managing…telling you what your priorities were going to be…telling us almost on a day-to-day or week-to-week basis what you’re going to be doing with your time and those kinds of things. We just kind of said, “No! No, that’s not the way it works.” And to some extent that worked. But they still control the purse strings and as such they still have a lot of kind of ‘leverage over’ – because they can always say, if you’re not going to behave the way we want you to, we’ll find another agency that will."

Importantly, funding arrangements varied in terms of how professional time could be flexibly deployed, for instance, for patient care, team meetings and collaborative case management. Revisioning the use of time was a critical strategy.

3. Revision use of time to meet the needs of client populations

Time and its use are crucial considerations, in terms of expectations regarding ‘how long’ it should take to see health improvements, and how providers use time. Working with populations whose health is so closely linked with social and economic conditions can seem slow or stagnant with respect to improvements in short- and long-term outcomes. Rather than constructing patients as ‘non-compliant’ or as failing to achieve goals, the Centre’s providers recognized how broader contexts influenced people’s health trajectories and decisions. The collective expertise of staff and interdisciplinary teams was used to determine when to support or ‘push’ patients to go a step further in moving toward their potential. This was particularly evident in relation to substance use and addictions issues, adhering to anti-retroviral treatments, safe-guarding against interpersonal violence, or working toward healthy parenting skills. Staff sought patient’s input about goals, discerning the patients’ readiness and supporting steps to prepare for change – a process that required long-term strategies and commitment. A patient described the impact of not “approaching me too fast”:

"What this place did for me was, actually, it really did help me out. Because it gave me a sense of security, a sense of trust with these people. And for me that’s a hard thing to do, just to trust people. Especially managers and staff members of any type….And staff members don’t approach me too fast, too hard and too quick, which is important, I believe. So, it’s, let me sort of come to you. And because it’s hard for me to ask for things…it’s hard for me to ask for help. So the staff here are patient with that. And I think they understand that, which is important."

Revisioning the use of time also refers to cultivating health care relationships over time. Time is required for participatory engagement of patients, and is fostered by adopting clinic structures that encourage ‘patient activation’, such as patient-initiated appointments scheduled on a drop-in or pre-booked basis. Such scheduling structures need to be creative, fluid, and innovative to accommodate people’s rapidly shifting priorities and concerns, as this provider explained:

"So someone might come in and you think that they are going to be here for their ear infection. But then, you get to know them on a broader level, so you can say like, "how are things going at home?” And to really be able to see people in an ongoing way, and say like, "so where are you living now? And you know and how’s it going?"… So then you can have the opportunity and the time and the resources to address those issues."

Revisioning time also means recognizing the complexity of the work involved in providing PHC to marginalized populations. Organizationally, this required recog nizing the need (and scheduling time) for interdisciplinary meetings; putting in place adequate time and processes to support staff to provide care to people who are highly traumatized; and providing flexibility in scheduling so that patients with greater needs are provided more time. This strategy in turn requires collaborative decision-making.

4. Engage in decision-making on the basis of critical analyses of power differentials, flattened hierarchies within interdisciplinary teams, and shared leadership approaches

To address the health effects of persistent power inequities, stigma, everyday social exclusion, and systemic discrimination experienced by many marginalized patients, organizations must first reflect critically, and purposefully, on power relations within their organization – in relation to interactions with patients, staff, management, the wider community, and other sectors and organizations. For service providers including receptionists, professional staff, and management, this requires critical self-reflection on how power differentials are managed, particularly with patients who may find it challenging to relate with or trust people in positions of authority [20,51]. A physician with years of experience working at one of the Centres described this process: 

"You’re trying to reflect on, you know, check my attitude: was I being condescending? And I’m sure many times I am, because you know, like, you slip into that role right? You’re the doctor."

Another physician described the approach she takes to engage in critical reflexivity:

"You have to look at the privileges that you gained as a consequence [of your professional status and social location], and that’s very uncomfortable for people."

At the organizational level, attention must be paid to power inequities that often shape staff dynamics. For example, engaging in team meetings and sharing leadership in ways that intentionally aim to flatten professional hierarchies among staff are critical and prerequisite to addressing power differentials in relation to patient populations. Team meetings or case conferences can be conducted in ways to signal all staff input is important – including reception staff who often have the most frequent contact with patients and play an influential role in shaping access to services. Scheduling team meetings at regular intervals also is essential to foster interdisciplinarity and comprehensiveness of services – and is critical to developing synergy among staff and within the organization about how to maintain equity-oriented strategies.

5. Tailor care, programs and services to the context of people’s lives (e.g., cultural, social, gender, and demographic contexts)

This strategy rests on the premise that PHC services need to be meaningful to the patient-population served. The notion of ‘tailoring’ builds on and extends the notion of patient-centred care, and refers to the adaptations that can occur when the social and cultural contexts of local patient-populations are taken into account in the process of delivering care. These adaptations are significant when working with patient-populations who, in the process of seeking health care, often encounter dismissiveness, stereotyping and negative assumptions related to poverty, racism, substance use and mental illness [19,20].

Depending on the local community context, and the populations served, tailoring that attends to the social and cultural context of people’s lives could include: attention to styles of communication (both verbal and non-verbal); efforts to provide services (where possible) in local languages; and adapting clinical practice guidelines in ways that align with the priorities of patients’ lives (which can be chaotic or stable at various times). Most often, responsiveness to social contexts requires interdisciplinary team-based responses; at the organizational level, this means ensuring that staffing levels are adequate to assist patients with, for example, completing lengthy disability forms, liaising with social-services, child protection services and other state authorities, and coordinating with other health care organizations.

At the level of patient-provider interactions, tailoring is essential to draw in, rather than limit access to care by marginalized patient-populations. For example, what might be seen as excellent interpersonal communication in one cultural context might be seen as discriminatory or alienating in another. At the two PHC Centres, initial greetings among people who identify as Aboriginal often incorporate the phrase, “where are you from?” to signal an awareness of the importance of place and community of origin as an essential aspect of one’s identity. Among some immigrant groups, however, this phrase can signal that one is viewed as ‘Other’. Similarly, when working with women with histories of trauma who are at high risk of experiencing re-traumatization during physical assessments, offering choices and easing into health care encounters can offer high impact ways of recognizing people’s vulnerability and foster trust as an essential component of access to PHC.

Openly discussing various approaches to harm reduction, including how to manage opiate prescribing practices when there are high rates of substance use among the patient population, is an example of tailoring that can take place at organizational and provider levels. These organizational-level discussions are critical when working with client populations with high rates of substance use or chronic addictions combined with multiple forms of discrimination; conveying acceptance and non-judgmental approaches are imperative to supporting people’s access to PHC services. As one patient explained:

"The whole thing of addiction is having people listen and not judging. And most doctors, I know, except for the select few that are here, they are all judging, very judgmental of addicts."

Organizational policies can be tailored to meet the needs of individual patients by implementing clinical practice guidelines that are flexible, dynamic and can be adapted to the person’s life context, personal circumstances, and highest priorities. For example, rather than beginning with their substance use issues *per se*, staff at the Centres working with mothers who use substances often began with the women’s top priorities – supporting women to increase contact with children apprehended by state authorities, or supporting women to parent their children to prevent child apprehension. As one provider explained:

6. Actively counter the impact of intersecting oppressions on health, access to care, and quality of life

"From a care provider perspective…if I can help you keep your five-year-old daughter from going to foster care, you know, that is very rewarding for me, if I could be part of that solution – and very meaningful for her, and more so for the five-year-old."

PHC providers and organizations need to be aware of how marginalizing practices and social exclusion operate in structures and institutions, including health care, thereby shaping people’s health care experiences and access to services; then, they can actively implement strategies to counter these barriers. Our data aligns with the growing body of evidence showing that disempowerment and alienation of marginalized groups in society are major obstacles to achieving health equity [1,22,40,52]. The positive impact of actively conveying respect and acceptance to individuals – and attending to power differentials, is highly significant, particularly for people who experience social exclusion on an everyday basis. A woman who is a patient shared the impact: 

"Because a person doesn’t have that courage, you know, they’ll sit there on the street thinking, it’ll be [such a huge thing to go in for health care]…That’s why I come – because they’ll let everyone in…You’re missing so much on that connection – not connected to anybody…Those kinds of just simple gestures…just that little bit of, you know, good human touch, once in a blue moon, can really help somebody get to the next step, right? Good human interaction and connections."

Our findings showed how countering racism, discrimination, stigmatization, and social exclusion can be achieved by conveying unconditional positive regard for patients regardless of their circumstances, and fostering the human dignity of all persons even in informal interactions in waiting rooms, at reception desks, over the telephone, or during clinical encounters. Affirming approaches can be ostensibly ‘small’ gestures, achieved through non-verbal communication, tone of voice, and other types of communication, as one provider described:

"I’ll stop and have a small conversation with the person, or I’ll say, you know, I’m really happy to see you here today, to let them know that they’re appreciated. Because other than that, people never see them [as people]."

Creating an accepting, non-judgmental PHC environment also manifests in the ways staff respond to patients’ expressions of frustrations or anger. This requires that staff continually reflect on the personal and social contexts which give rise to patients’ expressions of anger, as a physician narrated:

"I had a woman, 20-something, working the street, HIV positive, heroin-addicted, comes in very angry, hostile…I agreed to take her on for methadone and HIV care, and she started out really hostile, but she kept coming back. And then, after a year…she’s like, “no, I wouldn’t go anywhere else” and, “I’m so thankful for being here.” I just needed to accept that…she might be angry and hostile for a while but…eventually…the real person will emerge…"

At an organizational level, actively countering the impact of intersecting oppressions requires policies that support a low barrier health care environment wherein patients are, as one staff member described, “just allowed to be.” They do not have to transform themselves into something else to be seen as legitimate or credible.

One indicator of accessibility, especially for people who are marginalized or ‘hard to reach’, is when patients drop in or hang out at a Centre, without a specific appointment. A patient described the benefits:

"It’s more or less come in and have coffee, you know. You’re welcome. You won’t be chased out. And I feel comfortable coming here, and like I said, because I see a lot of my people here. And a lot of the staff, you know, talk to you…Like I said there are times where I don’t even need to be here, but I come here to talk to a friend or have a coffee, you know, and just go home."

Our observational data showed that these strategies had a visible impact; expressions of frustration or hostility by patients were infrequent, despite sometimes long wait times in waiting rooms, and despite some people being visibly under the influence of substances. On occasion, when some people’s behaviours approached a disruptive level, staff (usually receptionists) respectfully used communication styles to diffuse tensions and set limits, which were usually respected. This contrasted with our prior research in emergency departments where we observed security guards routinely deal with behaviours in ways that often resulted in escalating frustration and aggressive behaviours, and ended in dismissal or banning of patients and police involvement [19,20,53].

PHC organizations can also exercise leadership within their communities by increasing awareness of how racialization and discrimination operate in health care settings such as emergency departments, and in the wider community, and by working to counter such oppression. For example, a nurse at one of the Centres informed the media about methadone dispensing pharmacies that were exploiting vulnerable patients for economic gain; this action contributed to an investigation and policy changes to prevent such exploitation in the future.

7. Create opportunities to promote and foster engagement with community and other sectors, including participatory engagement by patients

Enhancing equity-capacity within PHC organizations ideally will involve engagement and collaboration with various sectors in the local community and beyond to maximize opportunities to address the social contexts of patients’ lives. For example, tailoring care, programs and services to the contexts of patients’ lives could involve liaising with child welfare agencies to develop ways to support women at risk of having their children apprehended. Actively countering discrimination could involve hosting an in-service at the emergency departments known to be frequented by patients to discuss possible anti-racist approaches. Recognizing that not all PHC organizations or staff have the flexibility to engage in these ways, this strategy is held out as an ideal to work towards, and as a starting point for meaningful dialogues within and between a range of health and social sectors. Such engagement also requires action at the level of policy, so that values related to equity can be effectively and democratically operationalized [54]. Following our example above, liaising with child welfare agencies to develop ways to support women would include, for instance, working on health policies related to child health assessments and social policies related to income support.

Participatory engagement of patients can take many forms and PHC organizations can play a role in providing a sense of meaningful belongingness, for example, by providing paid or volunteer opportunities including peer support programs. These experiences offer powerful points of connection for people marginalized by social and structural inequities, who often experience social exclusion. Having a meaningful focus and daily routine is particularly relevant for patients who are homeless or living in shelters, or who are unemployed or unable to be employed due to persistent chronic physical or mental illness, or substance use issues. At both Centres, for example, patients are periodically hired on a part-time basis (according to the availability of funds) to assist with peer support activities, focus groups, and women’s or men’s wellness programs. In one of the Centres, research funds for a self-management HIV program were used to hire patients as peer interviewers and research assistants. Patients often expressed a sense of ownership and responsibility in relation to ‘their’ Centre. In many cases, patients who used the Centres regularly played a role in shaping the physical and social milieu by voluntarily making coffee and replenishing supplies in the waiting areas, voluntarily helping with janitorial or maintenance tasks, and offering suggestions for how to make the physical space safer and more welcoming for women and children. Engaging in meaningful work-related activities through volunteer and temporary or occasional employment opportunities can be health promoting by enhancing people’s sense of self-efficacy, self-worth, and capacity to manage their health [55], as this patient described: 

"[I have] a paid position where I’m on call [for janitorial work]. And that helped me, kept me busy. It kept me from locking myself up and getting depressed…I’ve got something, like I’m doing something and I’m being responsible…If I didn’t have the volunteering I would have been lost…I would have just given up…Pay didn’t matter. It was just somewhere to go and know that I was needed, that’s what I needed, that’s kept me going."

Given the significance of participatory engagement, PHC organizations are well-justified in seeking or allocating funds to support volunteers or peer support workers. It is equally critical for PHC organizations to seek the input of patients at regular intervals so that their feedback is part of quality assurance, and to remain accountable to both local community and patient representatives by ensuring that the organization is welcoming and responsive to local populations. Organizations can build such approaches through policy. For example, one Centre requires that research conducted there include meaningful patient consultation and involvement in the research process, including employment (e.g., as research assistants or facilitators) when possible.

8. Tailor care, programs and services to the populations’ individual and group histories, with an emphasis on trauma- and violence-informed care

This strategy is based on the premise that it is critical to tailor programs and services to people’s individual and group histories. Because trauma and violence are intertwined with inequities, recognizing the significance of people’s histories means that PHC services must take into account the fact that most people affected by systemic inequities and structural violence have experienced varying forms of trauma. Additionally, because people with trauma histories often experience mistrust, building trust is critical to sustaining connections and a sense of personal safety in PHC [56]. As this patient elaborated: 

"We’re not just a number…That’s life saving, life changing, literally it’s that valuable, it’s life changing because when I was out there [on the street] it was not good. It was always for something, you know, you were selling your body or you’re giving up a piece of yourself daily. And then to have somebody want to do something for you, just to help you, like oh my god….It gives you a whole new perspective, it really helps."

Trauma- and violence-informed care is *not* about eliciting trauma histories; it is about creating a safe environment based on an understanding of the effects of trauma, so that health care encounters are safe, affirming and validating [24,29]. For example, PHC organizations serving high numbers of Aboriginal patients must recognize that many people have experienced the inter-generational effects of systemic and individual discrimination and racism, which can be conceptualized as one feature of historical trauma [21,22,57]. This conceptualization of trauma has implications for other groups of people, for example, refugees and in some contexts, new immigrants. In PHC contexts, such services are constituted by respectful, empowerment practices, not ‘trauma treatment’ such as psychotherapy (though referrals may be made for specific trauma therapy). For example, in recognition of the devaluing of Aboriginal culture as a result of Canada’s colonial history, one of the Centres featured signage in a local Indigenous dialect to convey a valuing of Aboriginal identity; in the other, a residential school healing program was implemented to support patients and their families.

The effects of trauma often are manifested in health and behavioural issues that initially appear to be unrelated [24]. Interpersonal violence experienced by women, for instance, is associated with acute and chronic issues such as: chronic pain (e.g., headache, migraine, back pain, pelvic pain, inflammatory bowel disease), arthritis, hypertension, higher rates of mental health problems, higher rates of substance use and dependence, and symptoms consistent with posttraumatic stress disorder (PTSD) [25,58]. Because these are common health issues, health services may fall short in integrating understandings of the long-term impact of trauma, and inadvertently fail to create an environment that validates patient experiences [24,25,30]. Trauma and violence-informed care, therefore, requires that PHC organizations integrate comprehensive and continuing education for all staff (including receptionists, direct care providers and management) about the health effects of trauma and the principles of trauma- and violence-informed care [59], and about strategies for actively minimizing the risk of re-traumatization in the everyday provision of services, as one health care provider explains: 

"So, [in case conferences and team meetings] we talk about…the level of trauma that this person comes from…Or how difficult it is for that person to walk through that door…Or how difficult it was for that person to actually bring up their concern to you…getting into conversations around our table about power imbalances and about how our interactions [with patients] can really affect this."

Equally critical are supports for staff who may be dealing with vicarious trauma when working with patient populations who have significant trauma histories. As described by one health care provider:

"She [was] a staff member that was not only in moral distress from what she was seeing in this community and taking it on but from also never resolving her own background trauma and seeing how it triggered her to work with the clients…but not having an employee assistance program [EAP] to refer her to for counseling [was hard]…I’ve lost about twenty [staff]…I’ve lost them…when they couldn’t take the stress…we don’t have any type of psychology services or EAP…"

Thus, tailoring and an emphasis on trauma- and violence-informed care must take into account the effects of working with highly traumatized patients, and as identified in strategy two, funding must be available to provide staff with adequate support. Given the correlation between trauma and all forms of inequity, trauma- and violence-informed care is also closely related to strategies to address social determinants of health.

9. Enhance access to resources that address the social determinants of health with an emphasis on advocacy and inter-sectoral collaborations

Addressing the social determinants of health is recognized as essential to PHC [7,38-40,60]; therefore, this strategy involves explicitly working to address these issues as legitimate and routine aspects of health care, often as the main priority. Maintaining or improving health is dependent on access to adequate safe housing, adequate nutrition, and meaningful activities. For example, mental health or well-being (especially in the face of PTSD and depression associated with all forms of trauma) is challenging to achieve in the context of inadequate social housing rife with rodent or insect infestations; physical health is challenging to achieve without a place to refrigerate food or medications [61]. At the individual level, this involves working with patients to facilitate access to social housing, food, and clothing, or supporting efforts toward paid employment. Our data repeatedly documented the impact of addressing people’s social needs as fundamental to maintaining or improving health, as this patient described: 

"I got the subsidy from them [the nurse and social worker at the Centre] for housing and I got the kids back now…I knew that I wanted to go to rehab [to address substance use issues]. I’ve been thinking about it for a whole year since I got the subsidy…That really made me look in the mirror at myself…[The subsidy] is like winning a lottery to me…[With] the subsidy, I thought, I’ve really got to smarten up, pull up my socks and start really watching the company I keep and start getting my life on track. And it’s getting there…."

Fostering connections with individuals, groups and resources is critical, because many people experiencing marginalization are disconnected from their families or home communities by policies, poverty and violence. Supporting connections to possible employment is critical because most people served cannot afford phones or computers, and thus have no way of being contacted.

At the organizational level, addressing this strategy involves leaders and staff within PHC agencies taking on advocacy roles and inter-sectoral collaboration in their wider communities to influence policies, such as working with housing organizations to maximize the availability of social housing; working with child welfare authorities to increase parents’ access to children and visitation rights; looking at ways to mitigate the root causes of homelessness; or advocating for more services for people with mental health problems. This attention to socio-political and economic environments must be extended to more immediate social and physical environments.

10. Optimize use of place/space to meet the needs of client populations

Increasingly, the socio-spatial environments of health care are being conceptualized as relational spaces that can be intentionally designed to support people’s subjectivities and experiences [62]. The notion of ‘therapeutic environments’ highlights the ways that socio-spatial environments can be transformed to foster people’s sense of agency and entitlement (or deservedness) when seeking PHC services. This has significant implications for patient-populations who experience discrimination and social exclusion in their everyday lives. A patient explained: 

"They make you feel welcome and they know you by name. And that means something to you, like somebody knows me, and it just makes me feel like a certain kind of pride inside you – like who you are and where you come from."

At the organizational level, this could translate into an agency’s commitment to create a low barrier and accepting environment, as a receptionist described:

"They come in and they’re cold and we welcome them with a warm coffee, and tell them they’re welcome to sit and chat, and sort of see how they are doing, where they slept, if they slept."

This contrasts with the atmosphere in conventional waiting rooms, as described by a physician:

"You know what [typical] waiting rooms feel like…that quietness, and contained-ness…Everybody sits in their little world. That is so foreign to our clients that they feel really out of place."

Essential to creating a therapeutic space are reception staff who play an active role in setting the tone in waiting rooms, during phone interactions, and during informal conversations with patients. This has implications for the kinds of training that staff receive, and requires their active involvement within organizational and decision-making structures. Creating supportive social spaces within PHC settings can involve, for example, having phones available for patients, a computer with internet access, and freely available coffee and tea. To fully address a population’s needs, providers may need to extend their influence beyond the boundaries of the physical PHC space by providing care in other spaces (i.e., shelters, people’s homes, single-room occupancy hotels, outreach services on the streets, etc.). Building on earlier strategies, patients can be integral partners in planning and creating such spaces and outreach processes.

In summary, these findings are based on a rigorous analysis of a large set of ethnographic data as the foundation for identifying the essential elements of equity-oriented PHC services when working with marginalized patient-populations, and will have broad application to a wide range of settings, contexts and jurisdictions. One limitation of this study is that the data collected were based on research conducted in only two PHC Centres in Canada. As well, the findings reflect team-based care as the primary mechanism for delivery of PHC services, and salaried versus fee-for-service compensation arrangements for physicians and other staff. Further research will be needed to examine the relevance and operationalization of these key dimensions and strategies when other models of service delivery are used, and in other jurisdictions and national contexts.

## Conclusions

A commitment to equity and social justice in PHC requires recognition of the particular health and social needs of people subject to systematic discrimination and relatively little power [63]. If the PHC sector is to become optimally relevant as a site for population health interventions, PHC organizations will need to prioritize locally-relevant strategies that are explicitly oriented to working with these groups, where it is expected that the greatest gains can be achieved.

If equity-oriented PHC organizations are to flourish, they will require support by inter-sectoral government policies and flexible funding models that recognize the essential role of community-based organizations in fostering health equity. The four key dimensions and 10 strategies for enhancing capacity for equity-oriented PHC services are important to hold out as ideals, given the persistence and increasing levels of health and health care inequities across population groups in Canada and other nations.

Recognizing that a wide range of models are currently used to address the health needs of vulnerable populations, consideration needs to be given to which aspects of the framework outlined in Figure 1 will be most relevant and feasible in particular contexts. The four key dimensions of equity-oriented PHC services are broad enough to be relevant as principles for guiding organizations and individual clinicians in primary care practices. Each organization, agency or practice can prioritize their starting point, given that the 10 strategies can be implemented incrementally, and in seemingly ‘small’ ways that do not require additional costs. For example, even at an individual physician’s practice, changing the manner with which receptionists answer phone calls or schedule appointments can have powerful effects on patients; in some settings, that kind of adjustment will signify that efforts are being made to counter the ongoing dismissal that many patients experience, thus addressing strategy six. Finding ways to address any aspect of the 10 strategies will have a synergistic and compounding effect because they overlap and intersect. The 10 strategies also can serve as a stimulus, for example, for developing new inter-sectoral collaborations; arguing for different funding models; expanding one’s referral patterns (e.g., to social service agencies, trauma-counsellors, etc.); and engaging in advocacy work or community service activities. Although some of the strategies may seem to run counter to dominant trends in health care that emphasize the rapid processing of patients, our research shows that if marginalized populations are to be served effectively, radically different approaches are required and can be legitimized as more efficient. These strategies also have the potential to positively influence PHC services more widely because by enhancing equity-competence for marginalized populations, improvements in PHC service delivery can be realized for all populations. Contrary to dominant rhetoric about efficiencies in health care, our data show that equity-based strategies can result in improved health outcomes and quality of life; the next step in research will be to link these strategies to quantifiable process and outcome measures.

## Endnotes

^a^ In Canada, the term ‘Aboriginal people’ is used generally to refer to Indigenous groups comprising First Nations, Métis and Inuit peoples [64]. These three groups reflect ‘organic political and cultural entities that stem historically from the original peoples of North America, rather than collections of individuals united by so-called ‘racial’ characteristics’ [64]^(p. xii)^.

^b^ PHC is conceptualized as the principal vehicle for the delivery of health care at the most local level of a country's health system, and is the first level of contact for individuals, families and communities, constituting the first element of a continuing health care process. The World Health Organization (WHO) claims that PHC embodies the principles of universal access, equity, and social justice [6,7]. Further, the WHO claims that effectively operationalizing PHC reform necessitates achieving universal access and social protection so as to improve health equity; reorganizing service delivery around people’s needs and expectations; securing healthier communities through better public policies; and remodeling leadership for health around more effective government and the active participation of key stakeholders. Implementing PHC is therefore foundational to addressing inequities, yet little progress has been made to date in actualizing that implementation.

^c^ Health equity is defined as the absence of systematic and potentially remediable differences in one or more characteristics of health across populations or population groups defined socially, economically, demographically, or geographically [7,11].

^d^ In Canada, "visible minority" refers to persons who are identified according to Canada’s Employment Equity Act “as being non-Caucasian..or non-white in colour. Under the Act, Aboriginal persons are not considered to be members of visible minority groups” [47].

## Competing interests

The authors have no competing interests to declare.

## Authors’ contributions

AJB, CMV, STW, VLS, JL, DL, DT, OG, MK, JO and PR conceived of the study. AJB, CMV, STW, VLS, & JL led all aspects of data collection, literature reviews, and collaboration with our clinical partners, and developed the initial draft of this manuscript. All authors participated in the analysis and interpretation of the results, and read and approved the final manuscript.

## Financial disclosure

This research was generously funded by the Canadian Institutes of Health Research Grant #173182. The funders had no role in study design, data collection and analysis, decision to publish, or preparation of the manuscript.
